# Photoluminescence Spectra of Helium Ion-Implanted Diamond

**DOI:** 10.3390/ma17215168

**Published:** 2024-10-23

**Authors:** Andrey A. Khomich, Alexey Popovich, Alexander V. Khomich

**Affiliations:** V. A. Kotelnikov Radio-Engineering and Electronics Institute of the Russian Academy of Sciences, Vvedensky sq. 1, Fryazino 141190, Russia; lex78@mail.ru (A.P.); alex-khomich@mail.ru (A.V.K.)

**Keywords:** diamond, ion implantation, helium, photoluminescence, Raman spectra, color center, defect

## Abstract

Ion implantation in diamond crystals is widely used both for producing conducting microstructures in the bulk of the material and for creating isolated photon emitters in quantum optics, photonics, cryptography, and biosensorics. The photoluminescence (PL) spectra of helium ion-implanted diamonds are dominated by two sharp emission lines, HR1 and HR2 (from Helium-Related), at ~536 and 560 nm. Here, we report on PL studies of helium-related optical centers in diamonds. Experiments have been carried out on a (110) plate of natural single-crystal type IIa diamonds. The uniform distribution of radiation defects in a 700 nm-thick layer was obtained by ten cycles of multiple-energy (from 24 to 350 kV) helium ion implantation with a total dose of 5 × 10^16^ cm^−2^. The diamonds were annealed in steps in a vacuum oven at temperatures from 200 to 1040 °C. It is demonstrated that helium ion implantation in diamonds followed by annealing gives rise to more than a dozen various centers that are observed in the PL spectra in the range of 530–630 nm. The transformations of the PL spectra due to annealing are investigated in detail. The spectral shapes of phonon sidebands are determined for the HR1, HR2, and HR3 bands with ZPLs at ~536, 560, and 577 nm, respectively, and it is shown that these bands are attributed to interstitial-related centers in diamonds. The reported results are important for understanding the structure and properties of helium-related defects in diamonds.

## 1. Introduction

Photoactive centers in diamonds are considered one of the most promising platforms for true single-photon sources and spin qubits, which are key elements of quantum communication, computation, and metrology applications. The bright and stable photoluminescence with high quantum efficiency and narrow lines of these point defects in the crystal lattice allow working with them at room and even higher temperatures, which cannot be achieved using most other quantum systems.

A diamond is a crystal characterized by extremely strong interatomic bonds. It has very low equilibrium solubility of impurities and their diffusion coefficients. Ion implantation and subsequent annealing is a standard procedure to introduce an optically active center into a diamond with a high degree of locality [1,2,3,4,5]. The most studied photoactive centers in diamonds are those containing nitrogen [6] and silicon [7]. Observation of two prominent peaks at 536 nm and 560 nm (HR1 and HR2) with a weak vibronic sideband was reported in 1983 in the cathodoluminescence (CL) spectra of helium ion-implanted diamonds [8]. Later, photoactive centers were discovered in diamonds, including germanium [9], tin [10], xenon [11], and magnesium [12] atoms, and their structure was studied. However, it has still not been possible to establish the nature of helium-containing centers in diamonds despite numerous studies [8,13,14,15,16,17].

Interest in the study of the behavior of helium in diamonds has been stimulated by the fact that along with hydrogen, helium as a light ion is widely used in fabricating diamond microstructures by ion implantation [18,19,20] and for creating vacancies in order to create shallow NV ensembles [21] for applications in ultra-sensitive, high-performance and innovative diamond quantum sensors (magnetometers, gyroscopes, spectrum analyzers, etc.). Hydrogen, which is chemically reactive and can easily diffuse along the implanted layer, is responsible for undesirable phenomena such as blistering and island graphitization after annealing [22]. Here, it is assumed that helium ions are chemically inert and do not noticeably change the photophysical properties of diamonds while creating an order of magnitude more vacancies than H+ ions [20]. It should be noted that when using helium implantation for the fabrication of color centers (NV, SiV, GeV, and others), there are optimal implantation doses that allow the creation of high concentrations of color centers while maintaining the structural perfection of the diamond crystal lattice [21].

An analysis of CL spectra of helium-implanted diamonds [13] and density functional simulations [23] showed that the crystal field in the diamond lattice is so strong that even noble gas atoms cease to be inert when inserted into certain lattice sites in diamonds and form covalently bound defects with the surrounding atoms, thus producing optically active centers. The formation of a chemical bond between an inert gas atom and a crystal lattice atom is explained in [13] by the size effect, i.e., by the fact that the difference between the covalent radii of the impurity atom and crystal lattice atom determines the parameters and properties of impurity-defect centers.

Spectral features with maxima near 536 and 560 nm, which are characteristic of helium color centers, were observed in the CL [8,13] and electroluminescence [14] spectra of single-crystal CVD diamond samples implanted with helium ions and annealed at 500–1200 °C. The HR1 and HR2 bands were observed in the CL spectra of ion-implanted (with energy of 10 keV) nanodiamonds [24]; this fact opens new possibilities for the practical application of helium-containing centers in diamonds. The intensities of HR1 and HR2 zero-phonon lines (ZPLs) as a function of the implantation dose and the temperature of subsequent annealing were studied in [15]; in [16], the authors studied the excitation PL spectra and emission lifetimes of helium-implanted (with energies of 1.3 or 1.8 MeV) synthetic single-crystal diamonds annealed at 1000 °C as a function of the measurement temperature.

However, it is still unclear how exactly the centers responsible for the HR1 and HR2 ZPLs arise and are transformed during annealing, what defects except helium are included in their structure, what is the spectral shape of the phonon sidebands of HR1 and HR2, what is the photostability of the HR1 and HR2 ZPLs, and what other helium-induced defects appear in the luminescence spectra of diamonds. Moreover, it has not been established yet whether the ZPLs at 560 and 536 nm are the fundamental and excited states of the same center, belong to different charge states of this center, or belong to different helium-containing defects in the diamond lattice.

The difficulties in studying helium-containing centers in ion-implanted diamonds and the corresponding HR1 and HR2 ZPLs are due to the low-intensity PL signal with a non-optimal signal-to-noise ratio and the spectral overlap of the phonon sidebands of the HR1 and HR2 ZPLs, both with each other and with the sidebands of the nitrogen-vacancy center (ZPL at 575 nm), which is characteristic of synthetic diamonds. The complex dependence of the ratio of the HR1 and HR2 ZPL intensities on the formation conditions of helium-containing centers in diamonds and the PL excitation parameters [15,16], as well as the different spatial locations of the maximum concentration of implanted helium and the region of maximum radiation damage in the bulk of the diamonds, also complicate the interpretation of the PL spectra.

## 2. Materials and Methods

To study helium-containing photoactive centers, a polished plane parallel disk with a diameter of 2.6 mm and thickness of about 0.14 mm was cut out from (110) plates of specially selected natural type IIa diamonds. Natural diamonds usually do not contain a single nitrogen atom in the substitutional position and, accordingly, do not have intense PL bands of NV centers in the green part of the spectrum, which spectrally overlap with the PL bands HR1 and HR2. This was confirmed by the measurements of both the PL and absorption spectra in the UV and visible parts of the spectrum. Nitrogen concentration in the form of A-centers in samples did not exceed 0.3 ppm as determined by measuring the optical absorption at 220–240 nm in the UV band [25] and from the IR absorption spectrum according to the procedure described elsewhere [26]. Transmission spectra were measured using a Specord M400 spectrophotometer (Carl Zeiss Industrielle Messtechnik GmbH, Jena, Germany) and a PerkinElmer Spectrum 100 FT-IR spectrometer (Waltham, MA, USA).

**Figure 1:**
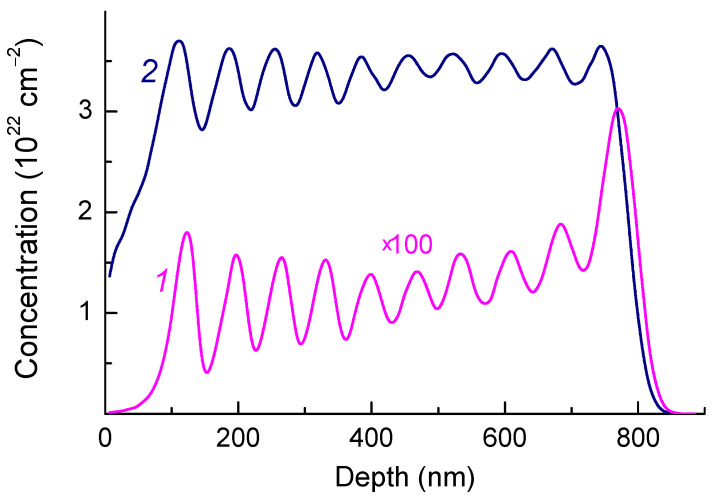
Profile of helium atoms (1) and vacancies (2) induced by He^+^ implantation with multiple energies and a total dose of 5 × 10^16^ cm^−2^ as a result of Monte Carlo (SRIM) simulation. The concentration of helium atoms is multiplied by 100.

The helium implantation was carried out by V. Dravin in the P. N. Lebedev Physical Institute on the heavy ion accelerator of “High Voltage Engineering Europe” (HVE, Amersfoort, The Netherlands). According to the literature [13,15,16] and our own data, a sufficiently high level of initial radiation damage is required to obtain samples with intense HR1 and HR2 ZPLs. The total implantation dose of helium ions was determined to be 5 × 10^16^ cm^−2^. To obtain a 0.7 μm layer of helium-implanted diamond with a uniform level of radiation damage throughout the thickness, ten cycles of implantation were performed with helium ions (energies from 24 to 350 keV). The helium ion doses for each cycle were calculated using the SRIM (Stopping and Range of Ions in Matter) program [27] (http://www.srim.org). The ratio of the number of implanted helium ions to the number of vacancies calculated by the SRIM algorithm is about 1:250 in our case (Figure 1).

To form an implanted area, we used a mask with a circular aperture with a 2.3 mm diameter which is smaller by 0.3 mm than the sample diameter. To reduce the recombination rate of Frenkel pairs, the implantation was carried out at liquid nitrogen temperature. Further details of sample preparation are presented in [28]. The properties of ion-implanted diamonds in the area of radiation damage in the structures manufactured using the technology [28] were uniform. This was confirmed by measurements of the surface swelling profile [28], uniformity of the implantation area color, and the invariance of the PL spectra when scanning the laser beam along the implantation area. The exceptions were natural diamond plates with a non-uniform distribution of impurity nitrogen concentration when implanted with doses comparable in magnitude to the nitrogen concentration. It is well known that nitrogen in diamonds actively interacts with radiation defects, which disrupts the recombination processes of Frenkel pairs, leading to an increase in the degree of initial radiation damage and, accordingly, to a decrease in the level of critical damage to the diamond [28]. The measurements here were carried out on type IIa diamond (impurity-free), and the implantation dose was close to the level of critical damage to the diamond, which ensured the homogeneity of the sample studied.

The vacancy concentration calculated by the SRIM program is usually used as an indicator of the level of radiation damage of diamond samples. It should be noted that the SRIM program does not take into account the interaction of defects with each other and their diffusion, as well as the phenomenon of the so-called “ballistic annealing” [29]. Nevertheless, calculations using the SRIM program make it possible to predict the doses and energies of ion implantation that lead to a critical level of radiation damage to the diamond and, hence, to its irreversible graphitization after high-temperature annealing, as well as provide information about the profile of radiation damage.

As a result of multiple-energy ion implantation, the deviation of the calculated vacancy concentration from its mean value in the helium-implanted layer of IIa diamond was at most 10% (Figure 1) and remained below the critical level throughout the implanted region. The implanted layer is covered on top with a thin (~0.08 μm) layer with lower radiation damage, which does not contain implanted helium; hence this near-surface layer does not introduce additional distortions into the PL spectra.

The helium-implanted diamonds were annealed in steps in a vacuum oven with graphite walls at temperatures from 400 to 1040 °C. After each annealing step, the PL and Raman spectra were measured on a Horiba Jobin Yvon LabRAM HR800 spectrometer (Lille, France) with a resolution of 0.5 cm^–1^ and excitation wavelengths of 473 nm (DPSSL KLM-457/SLN-50, FTI-Optronic, St. Peterburg, Russia) and 488 nm (Spectra-Physics Spectra-Physics, Mountain View, CA, USA).

## 3. Results

### 3.1. Raman Spectra of [He]-Implanted Diamond

**Figure 2:**
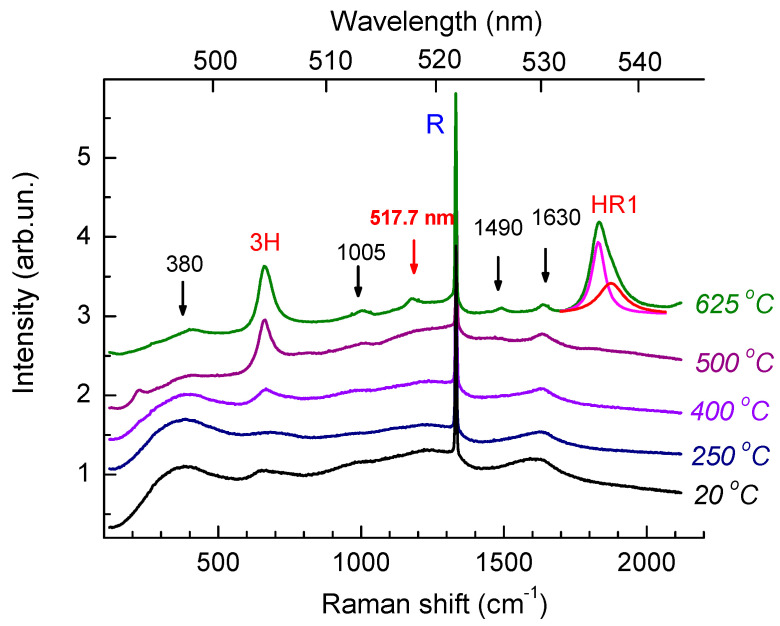
Transformations of Raman spectra of He-implanted diamonds with annealing temperatures. For clarity, the spectra are shifted vertically relative to each other. Black arrows indicate the Raman scattering bands, R is the Raman band of diamond, and the red arrow indicates a PL band with a ZPL at 517.7 nm. The top spectrum demonstrates the decomposition of the HR1 band into two Lorentzian components (pink and red). All the spectra are measured with excitation at 488 nm.

Raman scattering [30,31,32] and optical absorption [33] are an informative nondestructive methods for the analysis of radiation-damaged diamonds. Before implantation, the Raman spectra are dominated by an intense narrow peak of the diamond (Figure 2), which is detected against the background of a PL band with a maximum near 504.3 nm (at room temperature) that is associated with the H3 center consisting of a vacancy and two nitrogen atoms located in adjacent substitutional sites in the diamond crystal lattice [34].

The Raman spectrum of the diamond after multiple energy helium implantation corresponds to the spectrum of the diamond with high radiation damage (the bottom spectrum in Figure 2), as evidenced by the wide (FWHM~200 cm^−1^) low-frequency (maximum at 390 cm^−1^) boson peak [32], as well as an asymmetric peak with maximum at ~1600 cm^−1^, which is attributed to vibrations of several intrinsic radiation defects including C=C bonds in their structure [35]. It is believed that radiation defects of this type are precursors of graphitization in radiation-damaged diamonds. Disordering of the diamond crystal lattice due to the high concentration of defects leads to phonon confinement [35,36] or, otherwise, to a decrease in the mean free path of phonons to units of nanometers. The position of the peak with a maximum near 1600 cm^−1^ and the ratio of its amplitude to that of the boson peak [37] allow one not only to determine the critical level of radiation damage to the diamond but also to compare the levels of radiation damage as a result of ion implantation and irradiation with fast neutrons. The He^+^ implantation with a total dose of 5 × 10^16^ cm^−2^ (Figure 1) produces an amount of damage which, judged from the Raman spectra (Figure 2), is equivalent to the damage produced by fast neutron irradiation of 2 × 10^20^ cm^−2^. This is about 2–3 times lower than the critical level of radiation damage to diamonds.

**Figure 3:**
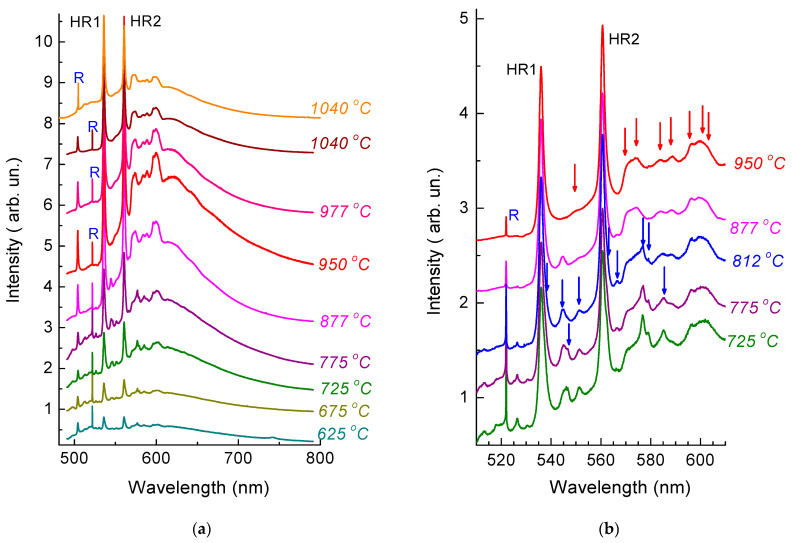
Transformations of PL spectra depending on the annealing temperature. For clarity, the spectra are shifted vertically relative to each other. All spectra in (**a**) are normalized by the integrated intensity of the diamond Raman band at 522 nm and at 503 nm in the top spectrum. The amplitude of the spectra in (**b**) is normalized by the amplitude of the HR1 band. Red arrows show spectral features associated with the HR1 and HR2 ZPLs, and blue arrows show the main spectral features associated with less thermally stable centers in the sample under study.

The diamond peak should have been absent in the Raman spectra of diamonds with such a high level of radiation damage [31,32]; however, we observe such a peak in the spectra of the sample under study (Figure 2 and Figure 3). We believe that the appearance of the diamond peak (FWHM~7 cm^−1^) in the Raman spectra of the sample under study is attributed to the less radiation-damaged near-surface layer with a thickness of about 0.08 μm (Figure 1).

The implanted layer acquired a brown color after ion implantation. Transmission in the green part of the PL spectrum (where the HR1 and HR2 bands are observed) decreased from 70% in the original diamond to approximately 20%. Such a change in the color of the initially colorless diamond corresponds to an absorption coefficient in a helium-implanted diamond layer of about 2 × 10^4^ cm^−1^, which corresponds to absorption in amorphized diamond. This confirms the Raman data on the high level of radiation damage to diamonds. The effect of helium implantation on the absorption spectra of diamonds in the entire UV-visible range was investigated in [28,33].

Figure 2 illustrates how the Raman spectrum evolves with annealing temperature *T*_ann_. As the annealing temperature increases, the band with a maximum at about 1600 cm^–1^ shifts to higher frequencies up to 1630 cm^−1^, narrows down, and decreases in intensity. Simultaneously a Raman band with a maximum at 1490 cm^−1^ manifests itself in the spectrum, which is characteristic of radiation-damaged diamonds and is attributed to Raman scattering by *π*-bonded di-interstitials [38]. The absorption coefficient in the visible part of the spectrum and the amplitude of the boson peak monotonically decrease with increasing annealing temperature (Figure 2), and the spectral feature of the phonon density of states (PDOS) of the diamond near 1000 cm^−1^ becomes more prominent in the Raman spectra. This points to a decrease in the degree of disorder in the material and the relaxation of elastic stresses in it. Transformations of the Raman spectra of radiation-damaged diamonds under recovery annealing are described in detail in [32].

### 3.2. Transformations of PL Spectra with Annealing Temperature

The recovery of the structure of the diamond crystal lattice gives rise to PL bands in the spectra. After annealing at 400 °C, the spectra (Figure 2 and Figure 3) clearly demonstrate a ZPL of the 3H center with a maximum at 504.4 nm (at room temperature), which is attributed to interstitial-related defects [38], while, after annealing at 625 °C, one can observe HR1 and HR2 ZPLs, as well as more than a dozen of narrow (FWHM~1 ÷ 2 nm) PL bands in the range of from 515 to 605 nm (Figure 3) with intensities 1–2 orders of magnitude lower than that of the PL bands HR1 and HR2. The bands HR1 and HR2 have an asymmetric shape due to the superposition of two closely spaced ZPLs. Figure 2 illustrates the decomposition of the band near 536 nm into two Lorentzian components (535.9 nm with FWHM = 1.8 nm and 537.1 nm with FWHM = 2.0 nm. After annealing at 625 °C (Figure 3), the band HR2 also consists of two components, 560.6 nm (FWHM = 1.9 nm) and 562.2 nm (FWHM = 1.6 nm). Similar doublets of HR1 and of HR2 bands were previously observed in the CL [13,25] and PL [16] spectra measured at cryogenic temperatures.

It is noteworthy that, in contrast to the band of the 3H center, the bands induced by the implantation of helium in the spectra in Figure 3 do not exhibit photochromism; accordingly, the differences in the spectra measured at different times in different regions of the sample are minimal. The most intense bands have maxima at 544.9, 546.5, 551.3, 566.5, 576.9, and 585.3 nm. Some of these PL bands with relatively weak electron–phonon interaction were previously observed in the CL spectra of He^+^-implanted type IIa low-nitrogen natural diamond (E = 80 keV, dose 2 × 10^16^ cm^−2^, T_ann_ = 500 °C)—the bands with maxima near 547, 551, 569.5, and 576 nm [13]; (E = 100 keV, dose 5 × 10^15^ cm^−2^, T_ann_ = 600 °C)—the bands with maxima near 546, 551, 576.5, and 585.5 nm [25], and in synthetic Ib diamond (E = 300 keV, dose 3 × 10^15^ cm^−2^, T_ann_ = 700 °C)—the bands with maxima at 546.5 and 551 nm [25]). The intensity of the PL bands of helium-implanted natural diamonds noticeably changes depending on the annealing temperature. Most of these bands correspond to color centers with lower thermal stability than that of HR1 and HR2. In Figure 3a, ZPLs of this type are indicated by red arrows. The relative intensity of the PL bands in Figure 3b changes with increasing annealing temperature, which may indicate the formation of several different color centers in helium-implanted diamonds. The same conclusion was made by the authors of [13], who measured the CL spectra of diamonds with different concentrations of nitrogen impurity at 100 K. Note that the set of bands in the CL spectra of helium-implanted HPHT and CVD diamonds is different from the bands observed in the spectra of natural diamonds. Usually, HPHT and CVD diamonds contain isolated nitrogen impurity atoms in substitutional sites, which, in turn, may form impurity complexes with intrinsic defects and/or with helium centers, as well as may change the position of the Fermi level in the forbidden gap of diamond, which may lead to a change in the charge state of the defect responsible for the color center.

The bands indicated by blue arrows in Figure 3b (in contrast to the HR1 and HR2 bands and their phonon sidebands—red arrows) almost completely disappear in the PL spectra as a result of annealing at 875 °C, thus presumably indicating the interstitial-vacancy annihilation mechanism. The intensity of the ZPLs at 537.1 and 562.2 nm significantly decreases with respect to the ZPLs at 535.9 and 560.6 nm after annealing at 950 °C; as a result, the HR1 and HR2 bands become symmetric and are well approximated by a Lorentzian shape.

The structure of the observed defects is still unknown; however, we assume that most of the narrow bands observed in Figure 3 are attributed to helium-containing centers in the diamond because, according to literature data, the PL bands with the same frequencies and FWHM have not been previously observed in the luminescence spectra of natural and synthetic diamonds, including diamonds irradiated with fast neutrons or implanted with other ions [25,39,40,41,42,43].

Radiation-damaged diamonds are characterized by optical spectra with a wide set of bands attributed to intrinsic defects, their complexes, and defect-impurity centers. The frequencies of these bands often coincide with those in ion-implanted and neutron- and electron-irradiated diamonds. Experimentally confirmed Monte Carlo calculations of particle trajectories show that, in typical ion-implantation regimes, atoms knocked out even by a light ion have enough energy to knock out new atoms. Therefore, the main radiation damage in diamonds implanted with ions with energies of several tens or hundreds of eV is caused by secondary collisions. For example, according to SRIM, the implantation of a single helium atom with an energy of 350 keV gives rise to 15 vacancies; i.e., the radiation damage pattern is determined by secondary intrinsic defects and their complexes. The main radiation damage to diamonds irradiated with fast neutrons with energy of hundreds of keV or several MeV is also due to the secondary processes of defect formation involving already knocked-out atoms, which form cascades of displaced carbon atoms. Therefore, for a high level of radiation damage, the Raman and IR absorption spectra of diamonds contain about the same set of intrinsic radiation defects irrespective of the radiation damage method [31,32,44,45,46,47]. The PL spectra of diamonds irradiated with fast neutrons or implanted with hydrogen isotopes with damage levels close to those in helium-implanted natural diamonds of type IIa (Figure 1) were analyzed in [43], where the authors observed only the H19 center with ZPL at 580 nm in addition to the well-studied NV (575 nm) and SiV (738 nm) centers. The H19 center is formed at high-temperature (1450–1650 °C) annealing and contains at least two vacancies in its structure [43], whereas 700–1200 °C annealing of diamonds irradiated with fast neutrons or implanted with hydrogen isotopes does not give rise to the bands observed in the PL spectra after helium implantation (Figure 3). This fact confirms the assumption that the PL bands in the range of 517–585 nm (indicated by blue arrows in Figure 3b) are also attributed to helium centers. One cannot rule out that some of the color centers annealed at T_ann_ ≤ 875 °C are precursors of HR1 and HR2 because the annealing of these centers at 800–950 °C leads to a simultaneous increase in the intensities of HR1 and HR2 ZPLs (Figure 3a). After annealing at 950 °C, the HR1 and HR2 reach the maximum amplitude with respect to the diamond Raman peak, as observed in the study of the CL spectra in [13]. Just as in [15], further annealing at 975 and 1040 °C leads to the quenching of the PL spectra; in this case, the shape of the PL spectra in the region of HR1 and HR2 ZPLs and of their phonon sidebands remains exactly the same as that after annealing at 950 °C.

Noticeable changes in the shape of the PL spectra after annealing from 950 to 1040 °C are observed only in the range of 502–506 nm, where the ZPL of 3H is detected, which is generally accepted as an interstitial-related radiation-related defect. According to the literature data, in type Ia diamonds, this defect can be annealed out at temperatures of about 600 °C, and in type IIa diamonds, it is annealed out at about 800–900 °C [48], whereas in a type IIb diamond sample (boron doped with p-type conductivity) heated to 1300 °C, this defect has much higher thermal stability [49]. The variability of the photo- and thermal stability of the center can be attributed to the change in the charge state of the defect responsible for this center [48]. The fact that the thermal stability of the 3H center in helium-implanted diamonds is close to that in type IIb diamonds (as also observed in [50]) may serve as indirect evidence of the fact that helium implantation gives rise to defects that can act as a mid-gap acceptor in the forbidden gap of the diamond. This conclusion is consistent with the results of density functional simulations [51].

As is known from the measurements of the CL spectra [13], annealing at 1200 °C leads to the complete disappearance of the HR1 and HR2 ZPLs from the spectra. To obtain a bright glow of helium-containing centers, one needs high doses of ion implantation at which a strong disordering of the diamond crystal lattice occurs. At the same time, according to the Raman scattering data [31,32], it is known that, with such a high level of radiation damage, the crystal lattice of diamonds irradiated with fast neutrons is not recovered after annealing at temperatures of about 1600 °C and even higher. Relatively low thermal stability in diamonds is characteristic of defect-impurity centers that contain interstitial sites rather than vacancies.

The top PL spectrum in Figure 3a was measured with excitation at 473 nm. The use of a different excitation wavelength did not give rise to additional bands in the PL spectra but led to an increase in the intensity of the HR1 band with respect to HR2. Such a variation in the ratio of the amplitudes of the HR1 and HR2 ZPLs is in agreement with the results of [16], where the authors investigated the PL emission intensity from HR1 and HR2 centers in diamond as a function of laser excitation wavelength in the 210–510 nm range. In [16], the authors found that the most efficient excitation of PL of the helium bands HR1 and HR2 occurs in the region of band-to-band transitions. An additional maximum of HR1 emission was observed at a wavelength of 490 nm; for the HR2 emission, there were two such maxima—at 438 and 458 nm. The 16% growth in the intensity of the HR1 band with respect to HR2 under the variation of the excitation wavelength from 488 to 473 nm (Figure 3a) is in good quantitative agreement with the results of [16].

### 3.3. Photoluminescence Saturation Properties of HR1 and HR2 ZPLs

**Figure 4:**
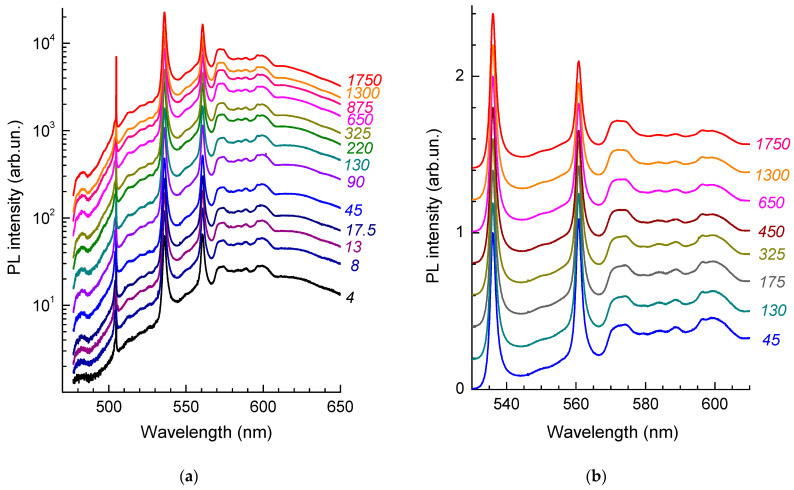
PL spectra as a function of laser excitation energy density (wavelength of 473 nm). The values of power density in kW/cm^2^ are indicated to the left of the spectra. The amplitude of the spectra is normalized (**a**) by the diamond band in the Raman spectra and (**b**) by the amplitude of the HR1 ZPL.

Room temperature photostability is an important parameter for the application of photoactive centers in diamonds in nanophotonics, quantum information processing, and metrology. We obtained a series of room temperature PL spectra at different laser beam power levels (Figure 4) to evaluate the saturation power for the HR1 and HR2 centers in helium-implanted diamond annealed at 1040 °C. The measurements were carried out at room temperature by a 473 nm wavelength laser beam focused to a 1 μm diameter spot in the power density range from 4 to 1750 kW/cm^2^. The laser beam intensity was reduced with the use of neutral-density filters integrated into the spectrometer. The amplitude of the Raman line with its linear dependence served as a criterion for the accuracy of power density measurement.

The amplitudes of the HR1 and HR2 peaks grow with increasing incident laser power somewhat slower than the amplitude of the Raman diamond peak; in this case, the peak HR1 reaches optical saturation slower than HR2. This is clearly seen in Figure 4b. For low values of the laser beam intensity, the amplitudes of the HR1 and HR2 peaks are close in magnitude, while, at P = 1750 kW/cm^2^, the peak HR2 is about 40% lower than HR1. The change in the intensity ratio of the amplitudes of HR1 and HR2 bands is not related to the heating of a sample in the region of laser beam focusing because an increase in the measurement temperature leads to the broadening and a long-wavelength shift of the maxima of ZPLs, which has not been observed in the experiment (Figure 4).

## 4. Discussion

### 4.1. Phonon Sidebands of HR1 and HR2 ZPLs

**Figure 5:**
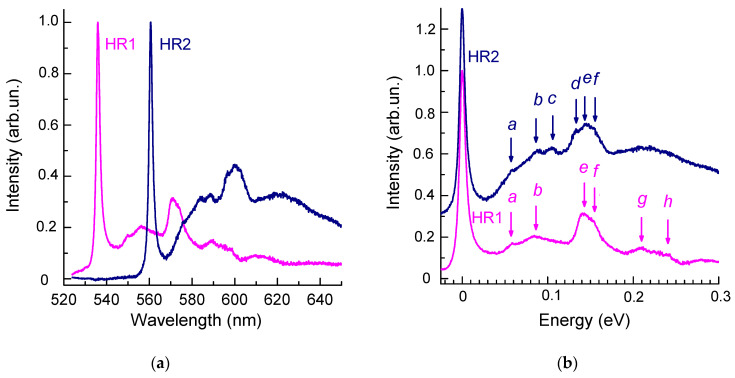
Result of decomposition of the PL spectra of helium ion-implanted type IIa diamonds annealed at temperatures above 900 °C into two components corresponding to the HR1 and HR2 bands. (**a**) The amplitudes of the spectra of HR1 and HR2 are normalized to unity. (**b**) Vibronic sidebands of the HR1 and HR2 ZPLs. For clarity, the positions of HR1 and HR2 ZPLs are shifted to zero energy.

One of the ways to study optical centers and crystal lattice dynamics is to study the structure of the phonon sidebands of the corresponding ZPLs in both absorption and PL spectra [25,52]. The difference between the spectral position of a ZPL and the features on the phonon sideband corresponds either to the frequencies of the vibrational modes at the critical points of the Brillouin zone or to the so-called local modes of defect and defect-impurity centers [52]. For HR1 and HR2, the difference in the spectral positions of ZPLs is 102 meV; i.e., it is less than the frequency of the diamond phonon in the Raman spectra and thus does not allow one to analyze separately the phonon sidebands of the HR1 and HR2 ZPLs. We used the significant difference between the dependences of the ratio HR1/HR2 in the PL spectra on the power density (Figure 4) and on the wavelength (Figure 3a) of the exciting radiation and spectrally divided the components of the spectra attributed to the HR1 and HR2 ZPLs according to the procedure described elsewhere [53]. This became possible due to the fabrication of a sample (Figure 1) with bright PL bands, a high signal-to-noise ratio, and good reproducibility of the PL spectra. The positions of the maxima and the FWHM of the HR1 and HR2 bands were almost independent of the laser beam power density (Figure 4), the wavelength of the exciting light (Figure 3a), and the region of the sample near which the measurements were carried out. All this allowed us to unambiguously distinguish the spectral shapes of the HR1 and HR2 ZPLs and the corresponding phonon sidebands. It is important to note that the results of the decomposition of the spectra measured with excitation at 473 and 488 nm, depending on the laser beam power density, were practically identical (Figure 5).

Figure 5b shows the spectra of the HR1 and HR2 bands the position of the ZPL, which is shifted to zero for clarity. The spectra of the phonon sidebands of the HR1 and HR2 ZPLs are similar in shape. This may support the hypothesis [25] that the HR1 and HR2 bands are attributed to the same defect.

The differences between the spectral position of the ZPL and the features on the phonon tail correspond either to the vibration frequencies at the critical points of the Brillouin zone or to the so-called local modes of impurity atoms forming the center [52]. Electron–phonon coupling of the HR1 and HR2 centers is characterized mainly by vibrations in the 130–160 meV range. The spectral positions of the features *d*, *e*, and *f* (Figure 5b) at 132, 144, and 155 meV (1065, 1162, and 1251 cm^−1^) in this interval well agree with the features on the spectra of the density of phonon states of diamond, namely, with TO(K), LO, LA(M), and LO(L) [25,54,55]. The feature *b* (87 meV or 702 cm^−1^) coincides with the frequency of a TA phonon near the minimum of the conduction band (at the point k_c_, k = 0.76 X) [56,57], and the feature with the energy coinciding with that of the feature *c* (106 meV or 855 cm^−1^) in the phonon sideband of the HR2 ZPL in diamonds was observed only in the absorption and luminescence spectra of the radiation-induced center 2.526 eV [25].

Diamond phonon density of states has no distinctive maxima and is rather low at energies below 70 meV and above 175 meV because the minimum phonon frequency in diamond at a singular point of the Brillouin zone is 71 meV (TA-phonon at point L), while the maximum frequency is 167 meV (Raman phonon at k = 0) [25]. Thus, we believe that the 58 meV (*a* in Figure 5), just as the 210 and 240 _M_эB (*g* and *h* in Figure 5b) vibrations, are quasilocal vibrations rather than lattice phonons. High-frequency quasilocal vibrations are characteristic of interstitial-related centers in diamonds [25,58]. For example, similar high-frequency vibrations, as well as quasilocal vibrations at 58 nm, were observed in the spectra of the 5RL centers with ZPL at 4.582 eV (270.5 nm). The 5RL center is a common radiation damage center, which is observed in the luminescence spectra of diamonds of various origins [58,59,60,61], and it is possibly produced by a <100>-split interstitial site [58,61].

Previously in [13], the authors observed a feature in the CL spectra of helium-implanted diamond that was located near 564 nm (2.169 eV), which is at a distance of 38 meV from the HR2 ZPL. On the basis of this observation, they made an assumption that there are helium-vacancy centers in diamonds. However, no features near 564 nm have been detected in the PL spectra of helium-implanted diamonds [15,16]; accordingly, the local vibration at 38 meV, characteristic of vacancies in the diamond crystal lattice [25], is absent in the PL spectra (Figure 3). It is likely that the feature observed in the CL spectra near 564 nm [13] is not associated with the system of HR1 and HR2 bands.

### 4.2. On the Interstitial Nature of He-Related Centers in Ion-Implanted Diamonds

The interstitial nature of the centers responsible for the HR1 and HR2 bands has also been confirmed by the dependence of their spectral position on elastic stresses. Radiation damage leads to the swelling of the diamond lattice [28,62] and to the corresponding shift and broadening of the Raman diamond peak and the characteristic peaks in the Raman scattering, absorption, and luminescence spectra. Tensile stresses in fast-neutron-irradiated or ion-implanted diamonds reach 10 GPa and even higher [28,62]. Subsequent high-temperature annealing partially restores the position of the maxima of the optical bands; however, at a high level of radiation damage, annealing at the maximum allowable temperature (at normal pressure) often does not guarantee complete restoration of the diamond crystal lattice and the return of the position and the FWHM of the optical bands to the values in undamaged or slightly damaged diamonds. For example, in diamonds irradiated with fast neutrons with fluences of 10^19^–10^20^ cm^−2^, the ZPL of the SiV center shifts to the long-wavelength side by 5–6 nm [63]. Such a significant shift in the ZPL maxima is characteristic of “soft” vacancy-impurity centers in diamond [64,65], whereas interstitial-related centers, as a rule, are classified as “hard” centers, in which the ZPL shift for the same values of mechanical stresses is several times smaller [58].

In [62], the authors investigated the transformations as a result of high-temperature annealing of the degree of volume swelling and the spectra of optical transmission of diamonds implanted with helium ions with energy of 350 keV at temperatures from 77 to 300 K. The measurements were performed on samples with damage levels both below and above the critical level. For diamonds with a radiation damage level close to that studied in this work, the swelling of the helium-implanted diamond decreased by approximately two times after annealing at 650 °C and by another two times after annealing at 900 °C [62]. The swelling of a diamond is accompanied by a change in the frequencies of the band maxima in both the Raman and luminescence spectra. Thus, the band 1490 cm^−1^ (Figure 2) shifts from 1490.5 cm^−1^ after annealing at 625 °C to 1496 cm^−1^ after annealing at 950 °C, which indicates the presence of elastic stresses in the sample.

**Figure 6:**
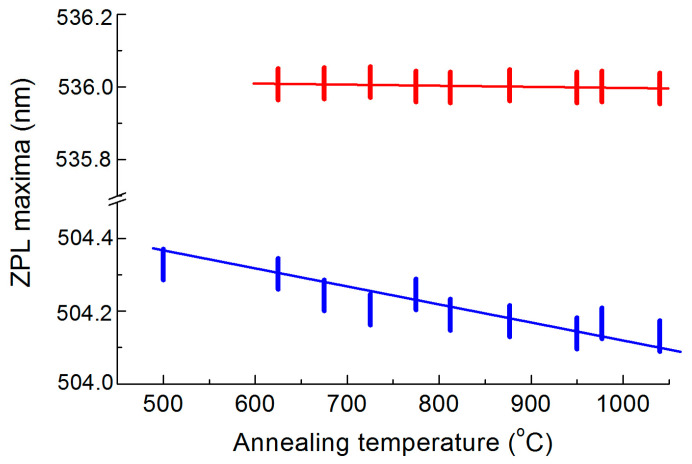
Positions of the maxima of the ZPL of 3H centers (blue pipes) and HR1 (red pipes) as a function of the annealing temperature of helium-implanted diamond. The pipe dimensions correspond to the error in determining the spectral position of the BFLs maxima.

In the PL spectra (Figure 3), the maximum of the HR1 band shifts by only 0.1 nm with an increase in the annealing temperature from 625 to 1040 °C (Figure 6). The position of the maximum of the HR2 ZPL was changed by approximately the same amount during annealing. Unfortunately, we could not estimate the magnitude of mechanical stresses from the changes in the position of the diamond Raman peak since the signal in the Raman spectra was formed in the slightly damaged near-surface layer of the diamond, which hardly contained implanted helium (Figure 1). Qualitatively, the degree of “hardness” of the centers responsible for the HR1 and HR2 bands can be judged from their comparison with the behavior of the ZPL of the 3H center in the same sample during annealing (Figure 6), which shifted by 0.3 nm in the annealing temperature range from 500 to 1000 °C. The positions of the maxima were determined by the approximation of the short-wavelength wing of the corresponding ZPLs by a Lorentzian shape. There are no quantitative data on the relationship between mechanical stresses and the shift of the ZPL of the 3H center in the literature. The interstitial-related center 3H [38] is a “hard” center in diamond; however, the centers responsible for the HR1 and HR2 bands exceed it in “hardness” by at least 2–3 times.

### 4.3. Saturation Pump Power of HR1 and HR2 ZPLs

**Figure 7:**
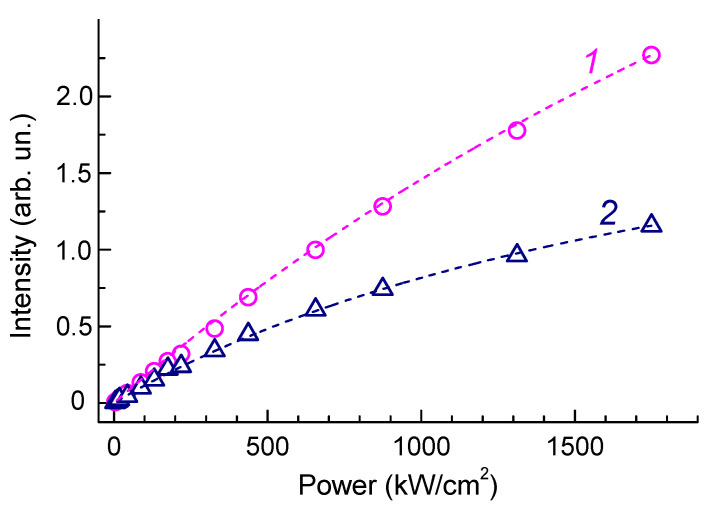
Integrated intensities of ZPLs for the HR1 (1, circles) and HR2 (2, triangles) centers as a function of the laser power. The dashed lines are the results of calculations for P_sat_ = 5000 and 2200 kW/cm^2^, respectively.

The results of the application of the decomposition analysis with respect to the segregation of the spectra of the phonon sidebands of the HR1 and HR2 ZPLs (Figure 5b) made it possible to analyze the dependence of the intensities of HR1 and HR2 on the laser beam power density at a wavelength of 473 nm (Figure 7) and determine the saturation pump power [66,67] for the ZPLs. The data are fitted well using Equation (1),

(1) $$I\left(P\right)=I_{\infty }\frac{P/P_{S}}{1+P/P_{S}},$$

where *I*_∞_ and *P_S_* are the saturation intensity and saturation power, respectively.

Based on the experimental data, we determined the PL saturation values for the HR1 and HR2 ZPLs to be 5000 kW/cm^2^ and 2200 kW/cm^2^, respectively. The high stability against laser excitation intensity is the advantage of the HR1 band. Figure 7 shows that almost no saturation of the HR1 band emission is observed at a laser power of 1000 kW/cm^2^, while for the NV and SiV centers, the critical laser intensities are an order of magnitude lower [53,68]. Note that the different dependences of the amplitudes of the HR1 and HR2 ZPLs on the PL excitation intensity may also be one of the reasons for the variability of the HR1/HR2 ratio in the measurements of the PL spectra, as was observed in [16].

### 4.4. Transformations of Less Thermally Stable Helium-Related Centers in the PL Spectra

**Figure 8:**
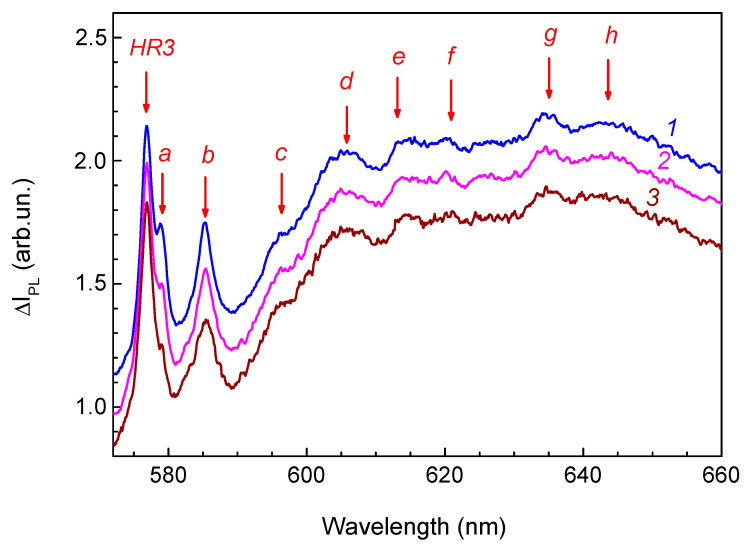
Difference PL spectra obtained by subtracting the spectrum measured after annealing at 977 °C from the PL spectra measured after annealing at 725 (1), 775 (2), and 812 °C (3). The amplitude of the difference spectra is normalized by the intensity of the band 576.9 nm (HR3). For clarity, the difference spectra are shifted in the vertical direction. All the initial spectra were measured under excitation at a wavelength of 488 nm.

The high “hardness” of the centers responsible for the HR1 and HR2 ZPLs and the invariance of the PL spectra after annealing at temperatures above 900 °C, combined with the optimal “signal-to-noise” ratio, allowed us to analyze in detail the behavior of other less thermally stable helium-related centers in the PL spectra of helium ion-implanted diamonds. To this end, we analyzed the difference PL spectra (Figure 8) obtained by subtracting with appropriate weighting factors the components due to HR1 and HR2 from the PL spectra.

**Figure 9:**
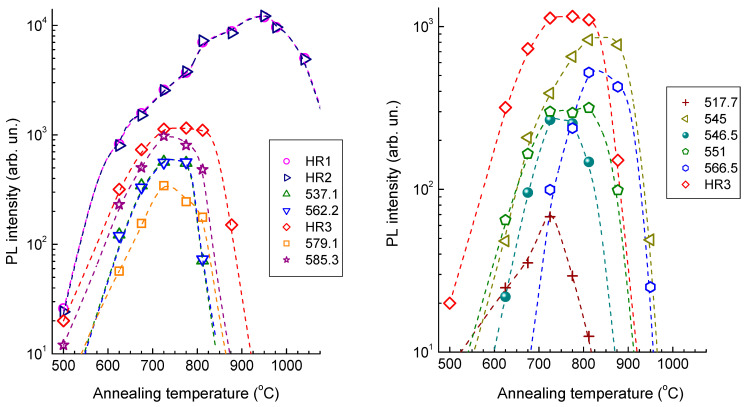
Integral intensities of individual ZPLs in the PL spectra of the sample of helium-implanted type II natural diamond as a function of annealing temperature. Dashed lines are guides to eyes.

A less intense and narrower band (FWHM = 1.5 nm against FWHM = 1.9 nm of the ZPL HR3) with a maximum near 579.1 nm (band *a* in Figure 8) is detected on the long-wavelength wing of the ZPL HR3, which is annealed at lower temperatures than HR3. The band with maximum at 585.3 nm (band *b* in Figure 8; FWHM = 2.3 nm) is also annealed at lower temperatures than the ZPL HR3 (Figure 9) in contrast to the bands *c*, *d, e*, *f*, *g*, *h*, and *i*, whose intensities in the range of annealing temperatures from 725 to 812 °C vary synchronously with the ZPL HR3. This suggests that at least part of these bands is due to radiation transitions of the center responsible for ZPL HR3 with the emission of phonons. According to the PL difference spectra (Figure 8), the lines at 576.9 nm (HR3), *c*, *d*, *e*, *f*, *g*, *h*, and *i* come from the same defect. The spectral distances between ZPL HR3 and the bands *c*, *d*, *e*, and *f* (70, 102, 125, and 152 meV) coincide with the position of singularities in the PDOS of diamond, namely, with phonon frequencies at the singular points of the Brillouin zone (TA(L), TA(X), LA(L), and LO(K), respectively [25,53,54,55]. The bands *g* and *h* (195 and 210 meV from the ZPL HR3) are characteristic of the phonon sidebands of centers with interstitials, for example, of 5RL [58,59,60,61] and 2DB [69,70]. The spectral position and the FWHM of ZPL HR3 are hardly changed under annealing, which testifies to the fact that this center is interstitial-related.

Thus, at least three groups of bands are observed in the range of 575–660 nm that are attributed to the centers formed in the type IIa diamond as a result of implantation of helium ions followed by annealing, namely, a system of bands of the center HR3 (576.9 nm) and the ZPLs 579.1 and 585.3 nm.

The rich structure of the PL spectra of helium-implanted diamonds (Table 1) can be attributed to the small radius of helium ions, which allows them to localize in various positions of the radiation-disordered diamond crystal lattice. According to [25], only in the range from 2.1 to 2.5 eV, the luminescence spectra of diamond samples of any type may contain up to 50 ZPLs after He^+^ ion implantation and subsequent annealing. In [51,71], the authors carried out ab-initio calculations for various helium-containing defects in the diamond crystal lattice; however, the results obtained turned out to be rather contradictory. One of the explanations for such a contradiction may be the fact that the formation of numerous bright PL bands requires a high level of radiation damage such that a set of various radiation defects is formed in diamonds [13,15]. This assumption is supported by the fact that the introduction of helium into the working medium during the CVD deposition of the diamond did not give rise to color centers but only led to a decrease in the size of crystallites with the formation of ultra-smooth diamond layers [72,73].

Figure 9 demonstrates the variation of the integral intensities of the main ZPLs observed in the PL spectra (Figure 3 and Table 1) relative to the amplitude of the diamond peak in the Raman spectra. HR1 and HR2 are the most intense and thermally stable PL bands in helium-implanted diamonds. The integral intensity of these two bands increases with increasing annealing temperature to a maximum value after annealing at 950 °C and then decreases (Figure 9). This behavior of the PL spectra is in good agreement with the data of other studies on helium-implanted diamonds in which the maximum intensity of the HR1 and HR2 bands was achieved in the CL spectra after annealing at 900 °C [8] and 1050 °C [13] and in the PL spectra after annealing at 1000 °C [15]. Small differences in the data of [8,13,14,15,16,25], in which implantation was performed with a fixed ion energy, can be attributed to both the differences in the energies and doses of implanted helium ions and the nonuniformity of the distribution profiles of intrinsic radiation defects and helium. The luminescence spectra of the samples prepared in this way contain bands of centers formed at significantly different levels of radiation damage.

The variation of the amplitudes of the ZPLs HR1 and HR2 occurs synchronously (Figure 9), which may support the assumption that these bands are attributed to radiative transitions of the same center. In the range from 700 to 950 °C, the rate of increase in intensity is approximately the same, with the exception of the region near 800 °C. Annealing at temperatures above 950 °C causes a synchronous decrease in the PL intensity of both bands and further annealing at temperatures up to 1200 °C leads to the rapid quenching of the luminescence intensity [13]. The intensities of two narrow (FWHM~2 nm) bands on the long-wavelength wings of the HR1 and HR2 ZPLs with maxima at 537.1 and 562.0 nm also vary synchronously; i.e., they shift relative to the HR1 and HR2 ZPLs by 1.3 and 1.6 nm, respectively. Both of these bands disappear in the PL spectrum after annealing at T_ann_ > 800 °C. The same bands were observed previously in the luminescence spectra of helium-implanted diamonds. For example, in the CL spectra of natural type IIa low-nitrogen diamond implanted with 100 keV He^+^ ions at a dose of 5 × 10^15^ cm^−2^ and subsequently annealed at 600 °C, the amplitudes of similar bands are close to those of ZPLs HR1 and HR2, and the additional ZPLs themselves are shifted by 1.3 and 1.5 nm, respectively [13]. The dependence of these two ZPLs on the long-wavelength wings of the ZPLs HR1 and HR2 on the implantation conditions and the annealing temperature, as well as their nature, was not studied and discussed in [13].

In the optical spectra of diamonds, due to the presence of narrow bands with FWHM 1–2 nm, closely spaced lines are often spectrally resolved. For example, in the PL spectra on the long-wavelength wing of the ZPL of the 3H center, the remarkably reproducible stepped satellite structure apparently associated with the 3H center [48] was observed, while the intensity of the narrow bands shifted by several nm from the ZPL of the 3H center was approximately a dozen times less than that of the 3H center. It was shown in the study of electron-irradiated diamonds [74] that these satellite lines are related to the 3H center and most probably originate from 3H defects interacting with some other point defects located in the vicinity of 3H at a certain distance from it. Such interaction leads to a decrease in the electron transition energy of the 3H center. We believe that such a model is suitable for explaining the behavior of the 537.1 and 562.2 nm ZPLs in the PL spectra of helium-implanted diamonds during annealing. Both of these bands can be attributed to optical transitions from the ground and excited states of the same center, which is simultaneously a precursor of the center responsible for the ZPLs HR1 and HR2. This is supported by the accelerated increase in the intensity of the HR1 and HR2 bands that occurs synchronously with the extinction of the 537.1 and 562.2 nm bands, respectively.

A comparison of the data presented in Figure 9 for the 544.9, 546.5, 551.3, 566.3, 576.9 (HR3), 579.2, and 585.3 nm ZPLs has shown that none of the pairs of bands exhibits absolutely synchronous variation in the relative integral intensities depending on the annealing temperature. We believe that almost all of the listed ZPLs belong to various different helium-containing centers in type IIa diamonds since similar bands have not been observed previously in the luminescence spectra of natural and synthetic diamonds [25,40,41], including radiation-modified ones, except the 546.5, 551, 577 (HR3), and 585.3 nm bands, which were observed in the CL spectra of diamonds implanted with helium ions [8,13,25].

This can be stated less confidently with respect to the weakest band at 517.6 nm since a narrow band with a very close frequency was previously observed in the low-temperature PL spectra of as-grown high-quality CVD synthetic diamond from Element Six under strong broadband (300–410 nm) UV excitation [42] and in electron-irradiated polycrystalline CVD type IIa diamond after high-temperature annealing of regions with an excess of B doping relative to N [48]. The appearance of the 517.6 nm band in the PL spectra (Figure 2 and Figure 3) may be attributed to the formation of radiation defects with acceptor properties in type IIa diamond as a result of helium implantation and subsequent annealing.

The presence of numerous lines in the PL spectra of diamonds implanted with large doses of helium should not be surprising. At a high degree of radiation damage of ion-implanted or fast-neutron-irradiated diamonds, several dozen lines corresponding to vibrations of various intrinsic defects and their complexes are recorded in their Raman spectra [31,32,45,75] and in the IR absorption spectra [25,44]. Some modes follow the same annealing curve, but more often, they anneal differently, indicating that it is a vibration of different defects.

Note that other bands were also observed in previous studies of luminescence spectra of helium-implanted diamonds, in particular, an intense band with a ZPL at 522.5 nm in the CL spectra, which was annealed at 850 °C [13]. However, such a band was recorded neither in our measurements (Figure 3) nor in the PL spectra [15,16] (the measurements were performed at excitation wavelengths of 532 and 405 nm) and the EL spectra [14]. According to [15], the source of PL at a wavelength of 522.5 nm in [13] could have been the diamond substrate rather than the layer implanted with helium ions.

## 5. Conclusions and Further Work

To summarize, we present an optical characterization of an ensemble of defects in helium-implanted type IIa diamond. The PL spectra measurements were carried out on a (110) plate of a natural single-crystal diamond. The uniform distribution of radiation defects in a 700-nm-thick layer was obtained by ten cycles of multiple-energy (from 24 to 350 kV) helium ion implantation with a total dose of 5 × 10^16^ cm^−2^. This made it possible to eliminate the superposition of signals from diamond regions with significantly different levels of radiation damage and to increase the signal-to-noise ratio in PL and Raman spectra measurements by at least an order of magnitude. Step-by-step annealing runs in the temperature range from 200 to 1040 °C made it possible to study in detail the transformations of defects induced by helium ion implantation into diamond. The maximum intensity of individual ZPLs in the PL spectra was achieved in the temperature range of 700–950 °C, and further annealing led to their quenching. The thermal stability of the most intense helium-related ZPLs decreased in the series (HR1 and HR2) > 544.9 > 566.3 > 576.9 (HR3) > 551.3 > 546.5 > 585.3 > 579.2 > (537.1 and 562.2). The variation of the amplitudes of the HR1 and HR2 bands occurs synchronously, which may support the assumption that these bands are attributed to radiative transitions of the same center. The same statement is true for the ZPLs at 537.1 and 562.2 nm, caused by a center that may be a precursor of the center responsible for the HR1 and HR2 bands. On the basis of the post-implantation thermal annealing behavior of the PL, we have concluded that the interstitials are involved in the formation of the HR defects associated with the ion implantation. The photoluminescence decomposition analysis has been successfully employed to determine the vibronic sidebands of the HR1, HR2, and HR3 ZPLs. We have shown that helium implantation gives rise mostly to acceptor defects. In general, the narrow emission of HR centers, their small phonon coupling and room temperature photostability (P_sat_ up to 5000 kW/cm^2^), the possibility of electrical excitation of HR centers [14], and the availability of largely accessible and easily focusable He beams for fabricating helium-related centers provide good prospects for the application of HR defects in quantum optics and photonics.

High-pressure high-temperature (HPHT) nanodiamonds, obtained by crashing bulk or micron-sized crystals, synthesized using conventional HPHT methods [76] or in a bottom-up HPHT growth process [77], are commonly used for use as optical biomarkers. Ion implantation into nanodiamonds is a well-established technology and allows obtaining nanosized (100–200 nm) diamonds with HR1 and HR2 bands in the PL spectra [24]. However, all types of HPHT diamonds usually contain relatively high concentrations of nitrogen in the substitution state, the presence of which significantly affects the luminescence spectra of helium-implanted diamonds [25]. In this regard, one of the tasks of future research is to study the processes of transformation of helium-related PL centers in HPHT diamonds containing nitrogen impurity.

Whilst this work reports many advances in the understanding of helium-related centers in PL spectroscopy, there are some areas that can be furthered to strengthen the assignments made here. As an extension, it would be interesting to investigate the temperature dependences of PL spectra, which will allow us not only to obtain information about the transformations of numerous types of defects formed in diamonds as a result of helium ion implantation and subsequent annealing but also to confirm the hypothesis that HR1 and HR2 are optical transitions from the excited and ground states of the same center. Further investigations are required to establish the He center’s structure and the site symmetry. Systematic studies of polarized luminescence of cubic crystals under polarized light excitation have been widely applied to establish the site symmetry of anisotropic defect centers [78]. Emission polarization spectroscopy has been proven effective in a number of cases for determining the symmetry and orientation of optically active defects in diamonds [79,80,81]; however, in the case of interstitial-related defects, such studies have been unsuccessful. The invariance of the spectral position and FWHM of the ZPL bands of the “hard” centers in helium-implanted diamonds during annealing also does not allow us to hope for determining their structure and site symmetry using uniaxial stress measurements. It seems that clear isotopic dependence would be a key characteristic for experimental investigations to elucidate the true structure of He-related centers in diamonds.

## Figures and Tables

**Table 1:** Spectral position of maxima and FWHM values for ZPL of helium-induced centers in diamond: our and literature data.

ZPL Maxima, nm	FWHM, nm	Data from Other Publications, nm
535.9 (HR1)	1.8	536.5 [13], 536.3 [14], 536.5 [15], 535.2 [16], 535.2 [25]
537.1	2.0	537.5 [13], 536.5 [25]
544.9	1.9	546 [25]
546.5	1.4	547 [13]
551.3	1.6	551.2 [25]
560.6 (HR2)	1.9	560.5 [13], 560.5 [14], 560.5 [15], 559.7 [16], 560.5 [25]
562.2	1.6	563.3 [16], 562 [25]
566.3	2.3	
576.9 (HR3)	1.9	576 [13]
579.1	1.5	
585.3	2.4	584 [13], 584.5 [16], 585.6 [25]

Ref. [13] cathodoluminescence spectra at 80 K, 2 × 10^16^ cm^−2^ [He], 80 keV, T_ann_ = 500 °C, Ref. [14] electroluminescence spectra at 300 K, 1 × 10^15^ cm^−2^ [He], 1.8 MeV, T_ann_ = 1000 °C, Ref. [15] photoluminescence spectra at 80 K, 1 × 10^16^ cm^−2^ [He], 1.3 MeV, T_ann_ = 1000 °C, Ref. [16] photoluminescence spectra at 25 K, 2 × 10^16^ cm^−2^ [He], 1.3 MeV, T_ann_ = 1000 °C, Ref. ([25], Figure 5.99, a) cathodoluminescence spectra at 80 K, 5 × 10^15^ cm^−2^ [He], 100 keV, T_ann_ = 600 °C.

## Data Availability

The original contributions presented in the study are included in the article, further inquiries can be directed to the corresponding author.
